# Communication Skills and Communicative Autonomy of Prelinguistic Deaf and Hard-of-Hearing Children: Application of a Video Feedback Intervention

**DOI:** 10.3389/fpsyg.2020.01983

**Published:** 2020-08-18

**Authors:** Meghana Wadnerkar Kamble, Christa Lam-Cassettari, Deborah M. James

**Affiliations:** ^1^School of Health Sciences, University of East Anglia, Norwich, United Kingdom; ^2^MARCS Institute for Brain, Behaviour & Development, Western Sydney University, Sydney, NSW, Australia; ^3^Education and Social Research Institute, Manchester Metropolitan University, Manchester, United Kingdom

**Keywords:** video feedback, prelinguistic, communication, autonomy, video interaction guidance, deaf

## Abstract

**Background and Aim:**

Evidence on the efficacy of parenting interventions to support communication development in deaf and hard-of-hearing children is emerging. In previous research, we showed that parental participation in a video feedback–based intervention enhanced parental self-esteem and emotional availability to their deaf and hard-of-hearing children. This paper investigates the impact of the intervention on the development of the children’s prelingual communication skills and autonomy. Evidence on the efficacy of parenting interventions to support communication development is warranted.

**Methods:**

Sixteen hearing parents with a prelingual deaf and hard-of-hearing child (*M*age = 2.05 years, SD = 1.77) were recruited by self-selection from pediatric audiological services and randomly stratified into intervention-first and waiting-list groups. Families completed three sessions of Video Interaction Guidance in their homes. Designed for maximal inclusion, the sample comprised children with complex developmental and social needs. The primary inclusion criterion was the child’s prelingual status (<50 signed/spoken words), which was established using speech and language therapy reports. Child communicative autonomy was assessed from a 20 min free-play video recording using a gold standard measure for deaf and hard-of-hearing children (Tait) before and after the intervention.

**Results:**

A Mann–Whitney *U* test indicated no significant difference between the two groups. The groups were collated, and a Wilcoxon signed-rank test with time (pre-/post-intervention) as a repeating variable was run. A significant increase in children’s communicative autonomy (*Z* = −3.517, *p* < 0.0001, *d* = 0.62) and decrease in children’s no-responses (*Z* = −3.111, *p* < 0.005, *d* = 0.55) were seen. There was no significant difference in the overall number of turn-taking between the parent and child, indicating differences in the quality of the parent–child interactions, not the quantity.

**Conclusion:**

This study adds to the emerging evidence for parenting interventions with deaf and hard-of-hearing children. We hypothesize that the video feedback intervention with its focus on emotional availability created space for the children to show increased communicative autonomy during parent–child interactions. Communicative autonomy is a long-term predictor of communication and linguistic development in deaf and hard-of-hearing children, and its conceptual underpinning makes it a good early measure of relational agency. Results can inform wider interventions that focus on the quantity of the parent–child communication.

## Introduction

Parent–child interactions provide a window into understanding the quality of parent–child relationships (Topping et al., 2013) and provide a pathway for the development of communication skills in the child (Bornstein, 2000). Emotionally attuned interactions marked with genuine warmth, sensitivity, and appropriate connectedness are essential for overall child development (Landry et al., 2006; Nelson et al., 2019). This includes the development of early brain systems (Piazza et al., 2020), social competence (Sheffield Morris et al., 2007; Rispoli et al., 2013), language (Topping et al., 2013), and cognition (Landry et al., 2003, 2006) in the short and long term (Nelson et al., 2019). A parent who responds to his/her child’s verbal and non-verbal communication cues in an attuned manner scaffolds the child’s communicative autonomy. Communicative autonomy occurs when an individuals can communicate their own intentions and motivations in a self-preferred manner (von Tetzchner and Grove, 2003). In the context of parent–child communication, communicative autonomy is created when the parent makes space for and responds to the child’s initiatives. The child can build upon these parental responses and create a reciprocal meaning-making environment (Troutman, 2015). However, there are several conditions where this communicative reciprocity can be challenged, which, when left unattended, can adversely affect the quality of the developing parent–child relationship (Easterbrooks et al., 2012). Research into the impact of a child’s disability on the parent–child relationship indicates that it is the complex interplay between the parent or caregiver’s psychological health and the nature of the child’s disability that affects the parent–child relationship rather than the child’s disability *per se* (Spiker et al., 2002; Howe, 2006). Parent training programs focused on improving the relational quality between the parent and the child can help mitigate risks associated with unhelpful parent–child communication (Letourneau et al., 2001).

Circumstances where a hearing parent has a child with congenital deafness provide a pertinent milieu to study the mismatch between communication demands resulting from the child’s hearing status (Barker et al., 2009). Universal Hearing Screening has significantly reduced the average age at which children with hearing impairment are diagnosed (Bamford et al., 2005). Still, disparities remain in the timely access of the intervention of choice, e.g., cochlear implant(s), for a number of families in many parts of the world, including the United States and United Kingdom, owing to a number of ecological factors such as hearing loss characteristics, parental demographics, and provider barriers (Hanvey et al., 2016; Bush et al., 2017). There is variability in the development of oral language even after 2 to 3 years of cochlear implantation even when a child’s age and implant use are accounted for Niparko et al. (2010), Svirsky et al. (2000), indicating that some children could be falling through the system and missing out on adequate support. The interaction of hearing parents with their deaf and hard-of-hearing children is known to be marked with more structuring and dominant interactions that are lower in sensitivity, responsiveness, and affect matching by the parent (Meadows-Orland, 1997; Pipp-Siegel et al., 1998; Lam and Kitamura, 2010, 2012), pointing toward an authoritarian parenting style (Knutson et al., 2004). Hearing parents are known to have fewer successful moments of interactions with their deaf and hard-of-hearing child (Beatrijs et al., 2019). Delays in communication (Barker et al., 2009), language (Moeller et al., 2007), and social competence (Hoffman et al., 2014) in deaf and hard-of-hearing children further compound the behavioral shortcomings on the parent’s part.

Exposure to a language-rich environment is essential for a deaf and hard-of-hearing child. Hence, evidence has successfully focused on promoting family-focused interventions (Yoshinaga-Itano, 2014). These interventions have focused on targeting “quantities” of a child’s speech and language outcomes such as speech perception, vocabulary size, and expressive and receptive language output (Svirsky et al., 2000), and on promoting higher “quantities” of parent language use such as conversational turns and mean length of utterances (Ambrose et al., 2014). Such quantitative indicators of improvement are receiving continued attention and are supported by developments such as the Language Environment Analysis system (LENA) (Ganek and Eriks-Brophy, 2016) in the typical development, developmental disability, and childhood deafness literature. LENA is a digital language processor designed to capture and analyze extensive amounts of verbal data and produce core language metrics such as the quantity of adult and child words and the number of conversational turns (Ganek and Eriks-Brophy, 2016). A recent study by Christakis et al. (2019) reported on a clinic-based multimodal intervention that used LENA. Their study used a combination of brief instructional videos presented via a smartphone app, advice from physician(s), and brief coaching based on the LENA counts to test whether an intervention improved home language environment and language development. The intervention consisted of short actionable tips and feedback to promote behavioral change in 61 parents of typically developing children aged 2 to 12 months in a pediatric clinic in a pre–post and follow-up design. Results at follow-up indicated significant improvements in adult word counts and parent–child conversational turns, but no improvements in child vocalizations (Christakis et al., 2019). In another study, Nilsen et al. (2016) examined the influence of para-linguistic factors and mother–child communication where the child had ADHD. Their results found that a child’s level of ADHD impacted on the mother’s para-linguistics factors including the pitch level and amplitude of the mother’s voice (Nilsen et al., 2016). It is important to understand how the style of parent communication and timing of speech input shape communication between hearing parents and their deaf or hard-of-hearing children. There is evidence that parallel talk or commenting, sensitivity of responding, and being child-led support language outcomes in deaf/hard-of-hearing children (Cruz et al., 2013; DesJardin et al., 2014), and these are usually suggested as means of supporting communication. In a qualitative research study, Decker and Vallotton (2016) interviewed 12 hearing parents of children with hearing loss to explore the nature of information that parents receive to help with management of their child’s hearing. The key theme that emerged was for parents to “keep talking” to their deaf and hard-of hearing child and to focus on sound and the child’s hearing. This advice to “keep talking” is common and an essential piece of advice in early interventions for deaf and hard-of-hearing children. However, if analysis of parent–child conversation is limited to “word counts,” the bidirectional reciprocity and characteristics of attuned parent–child interaction (Jaffe et al., 2001; Beebe et al., 2010) is largely overlooked. Both the “quantitative” and “qualitative” indicators are essential as measures for communication development and provide ways of scaffolding communication and language development, respectively. However, there is a danger that a focus only on the “quantitative” factors can miss the underlying fundamental relational and dialogic context of communication development. This relational and dialogic context is critical for language development in both children with and without hearing loss. Evidence suggests that early interventions and a focus on maternal sensitivity where parents learn to attune to the child can enhance language outcomes in deaf and hard-of-hearing children (Ching and Dillon, 2013; Quittner et al., 2013). Thus, it can be postulated that using relational principles of attending to and tuning into the deaf and hard-of-hearing child can potentially result in communicative advantages for the child. However, a gap remains in understanding precisely how interventions impact on the quality of vocal communication between hearing parents and their deaf and hard-of-hearing child (Cruz et al., 2013).

Tait et al. (2001) developed a video coding framework to measure early communication development in deaf and hard-of-hearing children. This measure examines the quantity and quality of turn-taking between the parent and child and classifies the child’s role in these turns as autonomous or not. The concept of communicative autonomy as used in this study is shaped and molded by the Tait measure. Past evidence using the Tait measures indicated that the level of communicative autonomy shown by deaf and hard-of-hearing children prior to cochlear implantation was related to performance on speech perception tasks post-implantation (Tait and Lutman, 1997; Tait et al., 2001, 2007). Another study that used the Tait coding framework as an outcome measure after cochlear implantation showed a quick increase in turn-taking and a slow increase in autonomy 12 months post-implantation (Chen et al., 2011). Other research in children with cochlear implants found a positive but weak relationship between prelinguistic communication and language development (Kane et al., 2004). These studies indicate that communicative autonomy can serve an important function in the development of speech and language skills and be a way of measuring early communicative development in deaf and hard-of-hearing children. However, these studies did not explore the quality of maternal sensitivity or communicative space making within the parent–child interaction.

Communicative autonomy indicates self-determination on the person’s part to operate and relate to others from either an instrumental (“*I–it*”) or a mutual (“*I–you*”) perspective (Zank and Braiterman, 2014). Buber’s relational ontology of dialog explains that the fundamental need of individuals is to relate to others, and it is this relational need that defines our existence (Zank and Braiterman, 2014). A highly autonomous individual will act in accordance to their authentic interests or values and shape the relational dynamics by creating and maintaining the interactional space (Ryan and Deci, 2000). Parenting practices that promote autonomous child behavior and experiences socially reinforce optimal communicative experiences for the child and provide a foundation for “relational agency” in the child. Relational agency occurs when individuals actively participate and contribute to their life circumstances within the context of their family and social life (De Mol et al., 2018). One can argue that in family communication, parent–child interaction is the space where relational agency is developed, as relationships are constructed in daily communications (Relational Dialectics Theory) (Baxter, 2011) and mediated by intersubjectivity (Trevarthen, 1979). In the childhood deafness literature, the Tait video analysis provides an opportunity to investigate communicative autonomy given its focus on *prelinguistic development* (Tait et al., 2007) and to postulate its theoretical relevance to relational agency. The Tait measure is thus used as a primary outcome measure in the present study. An investigation into the number and nature of turn-taking episodes provides an opportunity to examine the *quantity* and the *quality* of interaction.

Video feedback interventions, such as Video Interaction Guidance (VIG), can enhance the quality of a parent–child relationship (Kennedy et al., 2011). VIG is a strengths-based effective intervention, which builds positive relationships through filming, micro-analysis of, and feeding back on positive moments in the interaction (James, 2011). Three to four sessions of VIG in dyads with and without child hearing loss are known to have a positive impact on the parent–child communication and relationship (Juffer et al., 2005; Lam-Cassettari et al., 2015). Following participation in VIG, hearing children who had been adopted showed a decrease in disorganized behaviors and attachment styles (Juffer et al., 2005). Similar increases in maternal sensitivity were shown by Lam-Cassettari et al. (2015), in the context of childhood hearing loss and use of VIG. Mothers also showed appropriate structuring, decreases in hostility, and increases in their perceived level of self-esteem. Improvements were also shown in child responsiveness and involvement in mother–child interaction (Lam-Cassettari et al., 2015). The positive impact that maternal sensitivity and attentiveness has on social interactions with children in the context of hearing loss has also been shown in the longer-term language growth of children who are deaf and hard-of-hearing and not attending a video intervention service (Quittner et al., 2013).

Over a period of one and a half years (2010–2012), our research lab conducted the first ever trial of VIG using an *N* = 1 intervention design with 16 families, who were concurrently randomized and stratified in either a waiting-list or an intervention-first group (James et al., 2013; Wadnerkar Kamble et al., 2014; Lam-Cassettari et al., 2015). The program of research focused on increasing parental sensitivity and attention to the strengths shown in the parent–child relationship where the child was deaf and hard-of-hearing. Results showed a large effect on the Emotional Availability (EA) scales for both the waiting-list and the intervention group (Lam-Cassettari et al., 2015), indicating that the video feedback intervention enhanced parental sensitivity and attentiveness toward their deaf and hard-of-hearing children. The only other published study that has used VIG with families of deaf and hard-of-hearing children is a non-randomized clinical trial with case reports (dos Santos and Brazorotto, 2018). This study found post-intervention improvements in parent–child interactions as measured by an observation scale looking at the use of facilitative language strategies by the parents such as being child-led and using an expansive vocabulary. Children in the dos Santos and Brazorotto (2018) study were older than our sample. There is no indication of the children’s communication or speech and language status in their paper.

It is not well understood how interventions including video feedback can shape communication skills in the prelinguistic phase in deaf and hard-of-hearing children (Terlektsi et al., 2019). Evidence is required to ascertain the influence of video feedback intervention on the deaf and hard-of-hearing children’s communicative autonomy and the development of relational agency. The current paper builds on previously published results (Lam-Cassettari et al., 2015) by looking at the quantity and the quality of child communication. This paper investigates how the intervention influences the (i) communication skills of the children based on the counts of turn-taking and no-responses, i.e., the quantity, and (ii) the quality of parent–child interactions, i.e., the child’s autonomy.

The research question for this paper was: What are the effects of the video feedback–based intervention on the prelingual deaf and hard-of-hearing child’s (i) communication skills (turn-taking and no-responses) and (ii) communicative autonomy? This study hypothesized that the intervention would enhance the prelingual deaf and hard-of-hearing child’s (i) communication skills, i.e., increase turn-taking and decrease no-responses, and (ii) communicative autonomy.

## Materials and Methods

### Participants

Sixteen families with hearing parents and congenitally deaf and hard-of-hearing prelingual children were recruited by self-selection from the Nottingham pediatric audiological services. Families responded to information packs provided at the audiological management services between June 2010 and July 2011. All participants were of British origin, except one who was European and a non-English speaker. The researchers worked with an interpreter for all assessment and intervention visits with this family. The study inclusion criterion was the child’s prelingual status of <50 signed/spoken words (Clark, 1996), which was established from reports by the speech and language therapist. Owing to the heterogeneity in children who are deaf and or hard-of-hearing, this study had a mix of age range (mean age 2.03 years, *SD* = 1.94, range 0.6–6.10 years) and developmental ability. There is a paucity of research with children who have complex needs along with deafness/hearing impairment (McCracken and Pettitt, 2011). Hence, this intervention study was designed to be maximally inclusive of prelingual deaf and hard-of-hearing children who had additional developmental and social conditions as shown by 37.5% of this sample having complex needs. The majority of the children were male, i.e., 69%. This concurs with a higher prevalence of males in congenitally deaf children in general (Cremers et al., 1994). Table 1 summarizes the demographic details of the children. The study achieved 100% compliance and no attrition. Participants were compensated for their travel costs to attend the laboratory assessments. Although this study had a heterogeneous sample of children, there was no statistical difference between the groups in terms of sex, level of hearing loss, type of hearing prostheses, presence of complex needs, birth order, or child age at enrollment to the study.

**TABLE 1:** Demographic details of the deaf and hard-of-hearing children (*N* = 16).

**Demographic**	**Details**
Sex	11 male, 5 female children; 15 mothers and 1 father
Age: M (SD) Age range	2.3 (1.94) 0.6–6.10 years
Level of hearing impairment	14 profound, 2 moderate–severe
Type of hearing prosthesis and length	9 with cochlear implants (0–12 months of use); 7 with bilateral hearing aids
Complex needs	10 with no complex needs, 6 with complex needs (37.5% of the sample had complex needs)
Details of complex needs	1 × autism, 1 × severe ADHD, 1 × severe learning disability, 2 × preterm and associated delay, 1 × cytomegalovirus/global developmental delay
Birth order	6 × first born, 8 × second born, 2 × third born

### Ethics

This research program received ethical approval from the Nottingham University Hospitals Trust and the Derbyshire Research Ethics Committee, United Kingdom (NRES reference: 10/H0401/10), with continued approval to analyze and present data from the original study. Written informed consent was obtained from parents before stratification.

### Procedure

Families were randomly stratified (Altman and Bland, 2005) to the intervention-first group (IG) or the waiting-list group (WLG) based on the child’s age, sex, level of hearing loss, and additional needs by a research assistant not directly involved with the data collection. Families in the WLG had double baseline sessions, i.e., pre-intervention baseline 1 and pre-intervention baseline 2, and only one post-intervention session. Families in the IG had double post-intervention sessions, i.e., post-intervention 1 and post-intervention 2, and only one pre-intervention baseline.

Data were collected by MWK and CLC before and after the intervention in a purpose-built family room at the Child and Family Research lab in the University of Nottingham over a period of 1 year. Data collection involved parents completing questionnaires, video-recording 20 min of unstructured parent–child free play, and participating in semi-structured interviews. Details related to the procedure are described in Lam-Cassettari et al. (2015). Families completed three sessions of VIG in their homes.

One of the authors (DJ), who was trained in Tait analysis, coded the videos following the Tait protocol after interventions were finished and all data collection was complete. The Tait coding involved viewing the recording of the 20 min of free play twice to find the 2 min with the most successful sequence of communication. The selection of the two consecutive minutes from each 20 min recording of the parent–child play session at the lab was the best section of natural play as judged by the coder (as per the Tait protocol). Criteria for selection in this study entailed the overall quality of the interaction between parent and child, the degree of active participation from the child, and the responsiveness of the mother to the child’s initiatives. The selection was not subject to inter-rater reliability; only the coding of the turn types was. To establish inter-rater reliability of the coded segments, a research assistant who was also trained in Tait analysis coded 30% of the videos, which were randomly selected from the entire sample of 48 videos. Inter-rater reliability was measured using intraclass-correlations (ICC). There was very good agreement between the first and second raters as indicated by ICCs ranging from 0.85–0.87 across all turn types. Both DMJ and the research assistant were blind to the order of test sessions, and neither was involved in the data collection.

### Outcome Measures

#### Dependent Variable

##### Tait video analysis measure

The Tait video analysis procedure is an established video coding framework of pre-verbal communication in the childhood deafness literature and has a high inter- and intra-rater agreement (Tait et al., 2007). Coding is performed on 2 min of purposefully selected audio–video recordings of the child to assess pre-verbal communicative behaviors. The Tait framework categorizes the child’s communicative behaviors (gestures and vocalizations) into three behavioral codes: (i) turn-taking between the parent and the child (gestural or vocal), (ii) communicative autonomy, and (iii) no-response (Tait et al., 2007). (i) Turn-taking is coded first. Turn-taking is defined as when the child makes use of the opportunity to communicate. The parent creates this opportunity for the child when he/she says/does something or leaves a pause for the child to respond. Turn-taking also occurs when the child interrupts the parent’s communication. Turn-taking provides an indication of the *quantity* of interaction. This study coded the turns as gestural and vocal but used a combined score of these two as a turn-taking score. (ii) Communicative autonomy is coded by counting the number of turns in which the child’s communication could not be directly anticipated from the parent’s earlier turn. For example, a child may look away when the parent offers something, and pick up another item. Communicative autonomy results in a change of focus/direction of the interaction with the parent (Tait et al., 2001). Communicative autonomy provides an indication of the *quality* of interaction. (iii) No-response is coded where the child *does not* respond to the parent when there is a clear opportunity for a response from the child, for example, when the parent asks the child a question (Tait et al., 2007). In this study, the purposeful selection of a 2 min coding frame was from a 20 min free-play video recording of the parent and the child. This free-play recording was the same as that used in our previous published work (James et al., 2013; Wadnerkar Kamble et al., 2014; Lam-Cassettari et al., 2015), hence giving the same context of observation, albeit with a focus on the child’s voice.

##### Specific details about the Tait coding framework as used in this study

The children in this study were at a very early stage of linguistic development. They did not speak or sign in sentences. Turns, whether signed or vocalized consisted of single expressions. The details for coding, interpreting, and analyzing were as follows:

*Identification of 2 min of coding segment:* Identify the section of consecutive play and the start and end points to capture 2 min.

*Transcription:* Using broad orthographic transcription, transcribe each of the carer’s and the child’s utterances and behaviors during the 2 min selection.

*Identify turn types:* Go through each child’s turn and identify the turns and their type taken by the child. For example, in the following segment, the mother did not take any turn between the two turns by the child. Hence, this sequence of two separable events was coded as two turns—one gestural and one vocal.


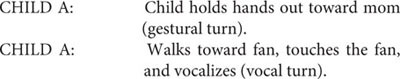


Identifying a non-response (a classified turn type) is easier than it might sound given that its classification is based on its absence. It occurs when the carer gives space for a response, expects a response, and no response is given by the child. This is exemplified as follows:


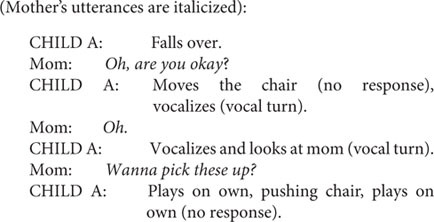


The above examples show how the child’s initiative and behavior were used to determine turn types, no-response, and gestural and vocal turns. The following is an example of communicative autonomy:


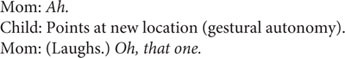


In this example, the child’s gesture has the result of directing the play as indicated by the mother’s response.

#### Quasi-Control Measure

##### Vineland Adaptive behavior scales

The Vineland Adaptive Behavior Scales (VABS) is a validated, reliable, and standardized developmental measure, with good internal consistency and test–retest reliability (Sparrow et al., 2005). The VABS can be used reliably with individuals with complex communicative needs and developmental delays (de Bildt et al., 2005). VABS was administered during a detailed parental interview (of 20–60 min duration) to assess the personal and social sufficiency of the child. The raw scores were converted to standard scores (*M* = 100, *SD* = 15). The Adaptive Behavior Composite (ABC) score was used to track developmental changes in the child before and after the intervention. The VABS was administered during pre-intervention baseline 1 and post-intervention 1 for both the WLG and the IG.

### Intervention

Video Interaction Guidance (James, 2011; Kennedy et al., 2011) is an evidence-based and accredited intervention using guided video feedback of spontaneous parent–child interactions to increase parental responsiveness to a child’s communication and behavioral cues and promote attuned interactions between parent and child. VIG involves an initial family-centric goal-setting session, which is followed by three goal-directed filming sessions and three shared review sessions of parent–child interaction in the family home. The shared review sessions are facilitated by a trained VIG guider (DMJ). Three short video clips (demonstrating attuned responses linked to the family’s goal) are played in each of the shared review sessions, and families are guided to microanalyze and reflect on the behaviors that facilitated successful communication with their child. The specific process of the intervention is described in detail in published work from our lab (Collins and James, 2013; James et al., 2013; Lam-Cassettari et al., 2015; James, 2017).

### Study Design

**FIGURE 1:**
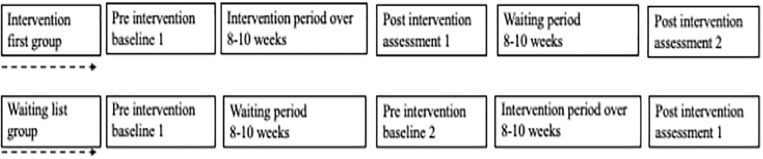
Study design.

**FIGURE 2:**
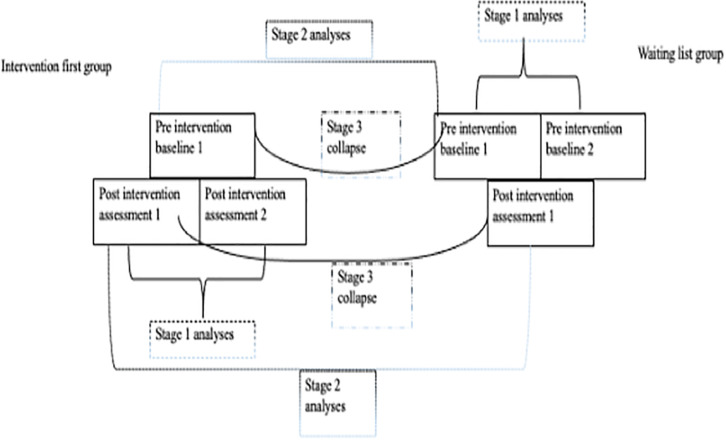
Illustration of the stage-by-stage process leading to the collapsing of the groups.

The original protocol from the larger research program employed double baseline for the WLG and double post-intervention sessions for the intervention-first group (IG) (Figure 1). This was to chart changes without the intervention and to capture the maintenance of any gains made during the intervention. The original protocol hypothesized that there will be no significant difference between the WLG and the IG. The groups were eventually collapsed to look at differences at pre- and post-intervention. The current study presents multi-stage analyses as shown below and explains the stage-by-stage process leading to the collapsing of the groups (Figure 2).

### Data Analysis

#### Stage 1 Analysis: Differences Within the Double Post-intervention Sessions for the IG and the Double Baseline Sessions for the WLG

A Wilcoxon signed-rank test was used to analyze differences within the double post-intervention and the double baseline sessions for the IG and the WLG, respectively. This was necessary to aid the choice of the very first pre (pre 1) and post (post 1) session for the stage 2 between-group analysis.

#### Stage 2 Analysis: Differences Between the IG and WLG at Pre 1 and Post 1 Intervention

A Mann–Whitney *U* test was conducted to test for between-group differences in the IG and the WLG at the pre 1 and post 1 intervention levels.

#### Stage 3 Analysis: Differences at Pre- and Post-intervention After Collapsing the Two Groups

The two smaller groups were collapsed to form one bigger group with time (pre/post) as the repeating factor in a within-subjects design. This was done to power the analysis and is the main analysis to address the hypothesis set for this paper.

A Wilcoxon signed-rank test was used to test for pre–post intervention differences. Spearman’s correlational coefficient was used to test for correlations between the Tait measures. Fisher’s *r*-to-*z* transformation was performed to compare the correlations.

Additionally, case-by-case scores are presented for the individual families to illustrate changes in the Tait measure at the pre- and post-intervention parent–child dyad level.

## Results

### Stage 1 Analysis

Differences within the double post-intervention sessions for the IG and the double baseline sessions for the WLG.

A Wilcoxon signed-rank test indicated that there was no significant difference within the double post-intervention sessions for the IG and the double baseline for the WLG. This determined the choice of sessions, i.e., pre 1 and post 1, that were included in the stage 2 between-group analysis. See Table 2 for the descriptive scores and statistics for the double baseline and double post sessions.

**TABLE 2:** Mean (SD) for scores from the Tait video analysis for the three behavioral codes from the original protocol for the intervention-first group (IG, *n* = 9) and for the waiting-list group (WLG, *n* = 7) for double baseline pre- and double post-intervention sessions.

	**WLG**			**IG**		
	**Pre-intervention baseline 1**	**Pre-intervention baseline 2**	**Post-intervention 1**	**Pre-intervention baseline 1**	**Post-intervention 1**	**Post-intervention 2**
Turn-taking	60.30 (17.69)	60.22 (14.62)	64.10 (17.02)	59.68 (20.63)	61.62 (16.06)	60.78 (14.88)
Child’s autonomy	9.22 (5.38)	15.91 (8.15)	26.53 (12.40)	14.51 (14.44)	33.06 (19.40)	32.61 (14.35)
Child’s no-response	25.47 (15.56)	24.57 (13.59)	6.85 (12.22)	20.76 (16.20)	1.74 (3.48)	1.96 (3.05)

*No significant difference within the post 1 and post 2 sessions for the intervention-first group (autonomy, *Z* = 0.943, *p* > 0.05; turn-taking, *Z* = −0.944, *p* > 0.05; child’s no-response, *Z* = −0.730, *p* > 0.05) and the pre 1 and pre 2 sessions for the waiting-list group (autonomy, *Z* = −1.85, *p* > 0.05; turn-taking, *Z* = −0.169, *p* > 0.05; child’s no-response, *Z* = −0.210, *p* > 0.05).*

### Stage 2 Analysis

Differences between the IG and WLG at pre 1 and post 1 intervention.

#### VABS

A Mann–Whitney *U* test showed that there was no significant difference between the IG and the WLG at pre 1 intervention in the ABC score or at post 1 intervention. See Table 3 for the descriptive scores and statistics for the VABS for the two groups.

**TABLE 3:** Mean (SD) for the pre- and post-intervention Adaptive Behavior Composite (ABC) score Vineland Adaptive Behavior Scales for the IG (*n* = 9), the WLG (*n* = 7), and the two groups as collapsed (*N* = 16).

	**Pre-intervention**	**Post-intervention**
IG	67.11 (23.12)	67.89 (29.40)
WLG	71.71 (5.31)	81.57 (14.25)
Collapsed groups	69.13 (17.38)	73.88 (24.32)

*No significant difference between the IG and the WLG at pre 1 intervention (*U* = 29.50, *p* > 0.05) and the post 1 intervention (*U* = 26.00, *p* > 0.05).*

#### Tait Video Analysis

A Mann–Whitney *U* test showed no significant difference between the IG and the WLG at pre 1 intervention for child’s autonomy (*U* = 29.50, *p* > 0.05), child’s no-response (*U* = 26.00, *p* > 0.05); and turn-taking (*U* = 31.00, *p* > 0.05). No significant difference between the IG and the WLG was seen at post 1 intervention for child’s autonomy (*U* = 27.50, *p* > 0.05), child’s no-response (*U* = 24.00, *p* > 0.05), and turn-taking (*U* = 29.00, *p* > 0.05). See Table 2 for the descriptive scores on the Tait video analysis for the groups. These results indicated that the two smaller groups could be collapsed into one bigger group.

### Stage 3 Analysis

Differences at pre- and post-intervention after collapsing the two groups.

#### VABS

A Wilcoxon signed-rank test indicated that there was no significant difference between the pre- and post-intervention ABC score (*Z* = −1.226, *p* > 0.05). See Table 3 for the descriptive scores on the VABS.

#### Tait Video Analysis

A Wilcoxon signed-rank test indicated that there was a significant difference on Tait pre- and post-intervention scores for child’s autonomy (*Z* = −3.517, *p* < 0.0001, *d* = 0.62) and child’s no-response (*Z* = −3.111, *p* < 0.005, *d* = 0.55). Turn-taking showed no significant difference (*Z* = −0.491, *p* = 0.623, *d* = 0.12). At post-intervention, there was a large increase in the median scores for child’s autonomy (pre Mdn = 8.50, post Mdn = 26.80) and a large decrease in child’s no-response (pre Mdn = 27, post Mdn = 0.00). Turn-taking increased slightly post-intervention (pre Mdn = 61.55, post Mdn = 64.50). See Table 4 for the descriptive scores on the Tait video analysis.

**TABLE 4:** Mean (SD) for scores from the Tait video analysis for the three behavioral codes of turn-taking between the parent and the child, child’s communicative autonomy, and child’s no-response, for the pre- and post-intervention sessions (*N* = 16).

	**Pre-intervention**	**Post-intervention**
Turn-taking	59.95 (18.77)	62.70 (15.97)
Child’s autonomy	12.20 (11.41)	30.21 (16.54)
Child’s no-response	22.82 (15.58)	3.98 (8.55)

At pre-intervention, a significant negative correlation was seen only between turn-taking and child’s no-response, Spearman’s *r*(16) = −0.498, *p* = 0.05, Fisher-*Z* = −0.547. At post-intervention, child’s autonomy was seen to be negatively correlated with turn-taking, Spearman’s *r*(16) = −0.844, *p* < 0.001, Fisher-*Z* = −1.23, and with child’s no-response, Spearman’s *r*(16) = −0.632, *p* < 0.01, Fisher-*Z* = −0.745.

##### Case-by-case investigation

**FIGURE 3:**
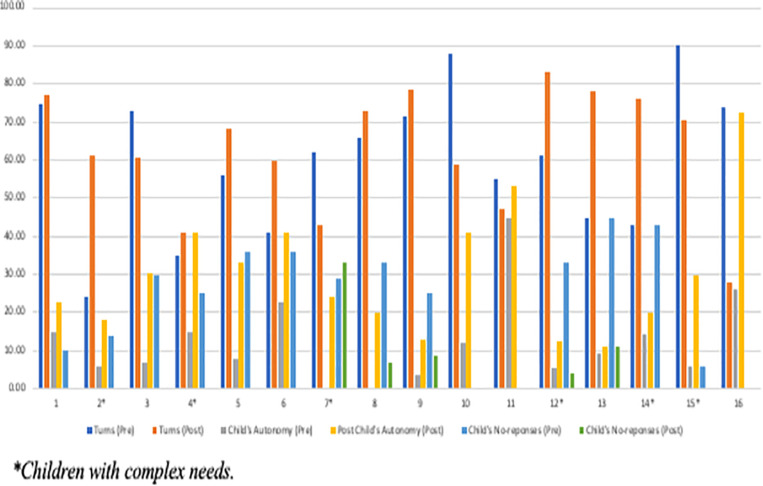
Case-by-case scores at pre- and post-intervention on the Tait video analysis.

A case-by-case investigation indicated that at post-intervention, all the 16 children had higher scores on child’s autonomy, turn-taking showed an increase in 10 cases, and 12 children had reduced no-responses. Six cases showed a decrease in turn-taking, and one child showed an increase in no-responses (case #7 with severe developmental delays). Three children (cases #10, #11, and #16, no complex needs) had the same number of no-responses (i.e., 0) at both pre- and post-intervention. These three cases are part of the six cases who showed a decrease in turn-taking at post-intervention. See Figure 3 for the case-by-case scores for pre- and post-intervention.

## Discussion

This study set out to examine the premise that parental participation in a video feedback–based intervention will enhance the prelingual deaf and hard-of-hearing child’s (i) communication skills, i.e., increase turn-taking and decrease no-responses, and (ii) communicative autonomy. Results partly support the hypotheses. We found a significant increase in child’s communicative autonomy, i.e., an indicator of the *quality* of parent–child interaction. Gains in the child’s communication skills were evidenced by the significant decrease in child’s no-responses. However, the number of turn-taking between the parent and the child did not change significantly. The *quantity* of parent–child interaction did change partly after the intervention, i.e., only a minor increase in the number of turn-taking, but no-response did decrease to a great extent. After the intervention, child’s communicative autonomy was negatively correlated with turn-taking and child’s no-responses. The strongest relation was seen between child’s autonomy and turn-taking. Non-significant results on the VABS and between the double baseline and double post-intervention sessions indicate that the children were not at an accelerated period of overall development. The general developmental changes, or the time lag between the sessions, cannot explain the changes in development of communication skills and communicative autonomy.

As demonstrated by the reduction of no-response, the deaf and hard-of-hearing children were making a lot more of their turns in relation to the space made by the parent for the child’s initiative. After the intervention, the parent–child interaction resembled more of a two-way dialogic interaction rather than the parent needing to keep creating opportunities for the child to participate. The interaction space between the parent and the child was being relationally shaped so that the deaf and hard-of-hearing child was an equal participant in shaping what was to happen next and not just a participant in keeping the conversation going. This *relational negotiation* could be what resulted in the difference between the quantity and the quality of the interaction. Based on the results of the correlations, the indicator of *quality*, i.e., communicative autonomy, was related to the indicators of quantity, i.e., turn-taking, and no-response, illustrating that the quality of parent–child interaction might move in the same direction as the quantity. Parents’ sensitivity of responding is known to support children’s language outcomes (Cruz et al., 2013; DesJardin et al., 2014). Video feedback–based intervention enhanced parental sensitivity and attentiveness (Lam-Cassettari et al., 2015), possibly leaving more space for the communicative ability and autonomy of the deaf and hard-of-hearing child to find their voice. This was true for almost all children in this study. However, one child with severe developmental delay had an increase in no-responses and a decrease in the overall number of turns. For this parent/child dyad, this showed an inverse relation between turn-taking and no-responses. Interestingly, three of the cases where the children were responding to their parent’s turns, i.e., had zero no-responses at pre- and post-intervention, showed a reduction in the parent–child turn-taking and an increase in autonomy after the intervention. This could support the inter-relatedness of the quality and quantity of parent–child interaction possibly mediated by parental sensitivity and attentiveness. If the parent occupies a more direct role in managing the turn-taking, this reduces the opportunity for the child to develop his/her autonomy in the interaction. In developmental research, the *qualities* of parents’ language are important along with the *quantitative* indicators. Just counting turns or utterances does not indicate the real changes that are happening with autonomy within dialog, i.e., relational agency. Previous studies did not explore the quality of maternal sensitivity or space making within the parent–child interaction. Investigations with hearing children have used a standalone coding frame to account for the children’s use of pointing and vocalizations and the mother’s attention. Such studies, which do include maternal responsivity, indicate a bidirectional nature of parent–child interactions and contributions of this interplay to subsequent language development (Wu and Gros-Louis, 2014). Scores on the Tait, a gold standard outcome measure, are known to be predictive of later speech and language development in prelingual deaf and hard-of-hearing children with cochlear implants (Tait and Lutman, 1997; Tait et al., 2001; Chen et al., 2011). Hence, the use of video feedback with hearing parents of deaf and hard-of-hearing children can be a way to bring about communicative gains and autonomy in the child.

Throughout their development, infants are given critical opportunities to learn as they partake in parent–child interactions in which parents provide opportunities for the child to develop his/her knowledge about the conversational rules and ways of relating to others (Laible, 2004, 2006; Laible et al., 2004). After the intervention, the hearing parent who is more attuned and attentive to his/her deaf or hard-or hearing child is creating communicative opportunities such that the child is getting to exercise his/her “dialogical agency.” Buber’s relational ontology of dialog can provide a framework to understand these results. Children could be relating as if they were aware of alternatives, making a transition between the *I–it* and *I–you* relations (Reddy, 2008; Lawrence, 2019) and thus beginning to exercise their “dialogical agency.” This could have been made possible by the increased sensitivity and attunement of the parents during interactions with their child. Based on the results from this study, it can be postulated that the child has increased agency after the intervention and is then influencing the parent more, and vice versa. The role and function of communicative autonomy in human interaction is beyond simply leading or being independent. The autonomous child could be acting with his/her interest and activating the parent to follow the child’s lead, thus making the deaf and hard-of-hearing child the agent driving the relational agency with respect to the hearing parent (Ryan and Deci, 2000). From the perspective of Self-Determination Theory (Deci and Ryan, 2012), an increased sense of autonomy serves a two-way function. It brings about a greater sense of integration within oneself, which in turn brings about a greater sense of relatedness with external partners, such as members of the family and society. The deaf and hard-of-hearing child could be finding ways to relate and respond better to his/her hearing parent as an equal and active communication partner rather than being solely reliant on the hearing prosthesis and communicative advances made by his/her hearing partner. In the Tait analysis, the pragmatic or communicative intent of the child overlays the modality of expression of the child’s turns. The overlaying of quantity and quality of the turn types as measured in the Tait analysis therefore represents the development of communicative intent and autonomy as it arises with and through the modality of expression. Communicative autonomy can serve an important function in relation making, and more specifically in the development of speech and language skills, indicating that interventions for promoting autonomy in parent–child interactions may lead to improved outcomes and should be implemented as early as possible after the diagnosis of deafness.

The video feedback–based intervention “VIG” is central to bring about this change (James, 2011; Kennedy et al., 2011). The intervention helps the parents to recognize and respond to the child’s emotional and behavioral cues and to re-establish or re-align the connection when required, i.e., increase attuned interactions (Doria et al., 2013). Attuned interactions can help to move the parent–child relationship toward intersubjectivity (Trevarthen, 1979). Intersubjectivity is when the relationship moves from the parent being a secure base for the child to a relational pattern where both the parent and the child are sharing experiences; developing understanding, knowledge, and expectations, i.e., both the parent and the child are socially constructing their relational space (Stern, 2005); and creating relational agency (Baxter, 2011).

In the field of childhood deafness, there could be a reliance on technology, e.g., cochlear implants, to bring about communicative gains. Giving access to technology does not result in the same gains in all children, especially for children with complex needs (Svirsky et al., 2000; Niparko et al., 2010). Our study found that working with parents does seem to change their approach to the communicative context (Lam-Cassettari et al., 2015). This could be the reason the children achieved a greater communicative gain in the current study. The Tait measure advances the tools used to capture changes in early communicative development and could be used in hearing difficulties/hearing dyads. Government policies on interventions to support deaf and hard-of-hearing children emphasize on the role of parents (Terlektsi et al., 2019). However, research has largely focused on parents’ role in shaping social interactions, much to the exclusion of the child’s role. Our research program was the first to start using video feedback (VIG) in working with hearing parents of deaf and hard-of-hearing children. The use of such video feedback to enhance a child’s communicative gains remains under-used in the field of childhood deafness. By means of the intervention, parents were guided to be attuned to the child and to give space for the child’s initiative, as indicated by the increase in the emotional availability scores, i.e., parental sensitivity and structuring (Lam-Cassettari et al., 2015). These parental behaviors might be supporting autonomy in the child. Based on the results, one can argue that parental warmth and reciprocity during free play will lead to more autonomy in communication.

## Limitations

The generalizability of these results is limited by the sample size, large standard deviations, and lack of a pure control group such as hearing children. Mediator analysis using the children’s age and a parental sensitivity measure such as emotional availability was not possible due to the sample size in this study. Future studies could include inter-rater reliability in the selection of the clips for coding. A bigger longitudinal study that follows the deaf and hard-of-hearing children for 24–36 months post the intervention will help provide definite results on the impact of the video feedback–based intervention and communicative autonomy on later language development. However, the current results suggest that improving communication through situated relationally based interventions could be an important factor to bring about changes at the parent and the child level. The increased autonomy could be predictive of later mastery of language irrespective of the modality of expression. A relational perspective should be integral to the early intervention strategies for prelingual deaf and hard-of-hearing children.

## Conclusion

This paper is important, as it highlights the importance of investigating the quality of parent–child interactions to communicative gains in deaf and hard-of hearing children using video feedback–based intervention, using an inclusive sample with complex needs. The development of communicative autonomy during the prelinguistic period is known to be predictive of later speech and language development in deaf children. It is the quality of the space that the parent makes for the child’s initiative that can shape the communicative environment. Thus, emotional availability of the parent is fundamentally important. Dialogue and participation in dialogue play a central role as the antecedents of language development. It is the deaf and hard-of-hearing child’s active involvement with meaning making and participation in dialog with the parent that creates relational agency.

## Data Availability Statement

The raw data supporting the conclusion of this article will be made available by the authors, without undue reservation.

## Ethics Statement

The studies involving human participants were reviewed and approved by Nottingham University Hospitals Trust and the Derbyshire Research Ethics Committee, United Kingdom (NRES reference: 10/H0401/10). Written informed consent to participate in this study was provided by the participants’ legal guardian/next of kin.

## Author Contributions

DJ was the project lead and created the theoretical rational for the project, carried out the intervention, and coded the behavioral data presented in the manuscript. MW and CL-C collected the data. MW collated and analyzed the data and wrote this manuscript. CL-C provided feedback and comments on the article. All authors contributed to the revision of the manuscript, read it, discussed and approved the final version.

## Conflict of Interest

The authors declare that the research was conducted in the absence of any commercial or financial relationships that could be construed as a potential conflict of interest.
